# Prognosis of Neonatal Arterial Ischemic Stroke

**DOI:** 10.15844/pedneurbriefs-29-5-3

**Published:** 2015-05

**Authors:** J. Gordon Millichap

**Affiliations:** 1 Division of Neurology, Ann & Robert H. Lurie Children's Hospital of Chicago, Chicago, IL; 2 Departments of Pediatrics and Neurology, Northwestern University Feinberg School of Medicine, Chicago, IL

**Keywords:** Cerebral Palsy, Epidemiology, Neonate, Outcome, Stroke

## Abstract

Investigators from University Children's Hospitals in Bern, Zurich, Aarau, and multiple other centers in Switzerland evaluated prospectively the epidemiology, manifestations, and treatment of all full-term neonates with neonatal arterial ischemic stroke (NAIS) and born 2000-2010.

Investigators from University Children's Hospitals in Bern, Zurich, Aarau, and multiple other centers in Switzerland evaluated prospectively the epidemiology, manifestations, and treatment of all full-term neonates with neonatal arterial ischemic stroke (NAIS) and born 2000-2010. The NAIS incidence in Switzerland was 13 per 100,000 live births. Median age was 2.0 days (range 1-26 days); median birth weight 3380 g (range 2370-4520 g). Of 100 neonates (67 boys) with NAIS all but 3 (97%) presented with seizures, and 50 (52%) had seizures as the only presenting symptom. Increased or decreased tone abnormalities were present in 32% and movement abnormalities in 11%; 81% had unilateral (80% left-sided) infarcts and 19% had bilateral lesions. The anterior circulation only (internal carotid, anterior cerebral, and middle cerebral arteries) was affected in 89%. Risk factors for NAIS were maternal risk conditions (32%), birth complications (68%), and neonatal comorbidities (54%). Genetic testing abnormalities included factor V Leiden mutation in 5%, heterozygous prothrombin mutation in 11%, and heterozygous methylene tetrahydrofolate reductase mutation in 36%. Seventeen percent received antithrombotic and antiplatelet therapy without serious side effects. At aged 2 years follow-up, 39% were diagnosed with cerebral palsy, 7 (9%) were treated for epilepsy (4 had infantile spasms), and 31% had delayed motor development. Children with normal mental performance at 2 years after birth may develop deficits later in life. [1]

COMMENTARY. A comparison of the incidence of NAIS in this population-based, prospective study with that in 9 previously published studies, 5 of which were population-based and 4 were hospital-based [1], the incidence varied widely from a low of 5 per 100,000 live births (a population-based study) to a high of 43 per 100,000 (a hospital-based study). The greater susceptibility of boys to NAIS is unexplained; boys and men have a higher incidence of stroke through life, and elevated testosterone levels increase the risk of cerebral thromboembolism [2].

Neonatal seizures are most commonly the clinical finding that triggers assessment in neonates with stroke. In children with NAIS without early-onset seizures, perinatal stroke is recognized retrospectively, with emerging hemiparesis or late-onset seizures presenting >14 days after the stroke. Risk factors for perinatal stroke include hereditary or acquired thrombophilia and environmental factors [3].
